# The association between gout and radiographic hand, knee and foot osteoarthritis: a cross-sectional study

**DOI:** 10.1186/s12891-016-1032-9

**Published:** 2016-04-16

**Authors:** Megan Bevis, Michelle Marshall, Trishna Rathod, Edward Roddy

**Affiliations:** Arthritis Research UK Primary Care Centre, Research Institute for Primary Care and Health Sciences, Keele University, Keele, Staffordshire ST5 5BG UK

**Keywords:** Crystal arthropathies, Osteoarthritis, Epidemiology, Radiology, Primary care rheumatology

## Abstract

**Background:**

Gout is the most common type of inflammatory arthritis and is largely managed in primary care. It classically affects the first metatarsophalangeal joint and distal peripheral joints, whereas the axial joints are typically spared. The reason for this particular distribution is not well understood, however, it has been suggested that osteoarthritis (OA) may be the key factor.

One hypothesis is that there is an association between the disease states of gout and OA as the conditions share common risk factors. The objective of this study was to determine whether there is an association between gout and radiographic osteoarthritis (OA).

**Methods:**

A cross-sectional study was nested within three observational cohorts of people aged ≥50 years with hand, knee and foot pain. Participants with gout were identified through primary care medical records and each matched by age and gender to four individuals without gout. The presence and severity of radiographic OA were scored using validated atlases. Conditional logistic regression models were used to examine associations between gout and the presence, frequency and severity of radiographic OA at the hand, knee and foot and adjusted for BMI, diuretic use and site of joint pain.

**Results:**

Fifty-three people with gout were compared to 211 matched subjects without gout. No statistically significant associations were observed between gout and radiographic hand, knee or foot OA. However, individuals with gout had increased odds of having nodal hand OA (aOR 1.46; 95 % CI 0.61, 3.50), ≥8 hand joints with moderate to severe OA (aOR 3.57; 95 %CI 0.62, 20.45), foot OA (aOR 2.16; 95 % CI 0.66, 7.06), ≥3 foot joints affected (aOR 4.00; 95 % CI 0.99, 16.10) and ≥1 foot joints with severe OA (aOR 1.46; 95 % CI 0.54, 3.94) but decreased odds of tibiofemoral (aOR 0.44; 95 % CI 0.15, 1.29) or patellofemoral (aOR 0.70; 95 % CI 0.22, 2.22) OA in either knee.

**Conclusion:**

There was no association between gout and radiographic OA, however, people with gout appeared to be more likely to have small joint OA and less likely to have large joint OA.

## Background

A link has been established between gout and OA, which could explain the distribution of joints affected by gout, particularly the tendency for the first metatarsophalangeal joint (1^st^ MTPJ) to be affected [1]. Previous studies have shown that joints affected by acute attacks of gout are more likely to display clinical or radiographic features of OA [2, 3]. This is particularly true for the 1^st^ MTPJ, mid-foot, knees and the distal interphalangeal finger joints [2]. Furthermore, monosodium urate (MSU) crystal deposits at the tibio-talar joints have been shown to occur at sites of degenerative cartilage lesions [4]. A recent primary-care based case–control study however, did not find evidence between gout and clinically-assessed nodal OA, although people with gout were more likely to have hallux valgus and chronic pain in the knee and big toe, compared to age- and gender-matched controls without gout [5].

The aim of this study was to investigate the association between gout and radiographic OA by comparing the presence and severity of radiographic hand, knee and foot OA in adults with and without gout.

## Methods

### Study population

The study used baseline data from three prospective population-based cohorts of people aged ≥50 years with pain in the hand, knee and foot: Clinical Assessment Study of the Hand (CASHA) [6], Clinical Assessment Study of the Knee (CASK) [7], Clinical Assessment Study of the Foot (CASF) [8] respectively.

Each cohort invited participants to complete a self-reported postal questionnaire. In CASHA, CASK, and CASF, those reporting hand, knee, and foot pain respectively in the last year were invited to attend a research clinic. Radiographs of the hand and knees were taken in CASHA and CASK; radiographs of the hands and feet were taken in CASF [6–8].

### Participants with gout

Participants with gout were identified through primary care medical records using specific Read codes for gout in the period from 18-months before to 18-months after clinic attendance. Individuals without a Read code for gout but who had “gout” mentioned in their consultation free-text were also identified and included if there was mention of a gout flare/attack or clinical features suggestive of gout.

### Participants without gout

Each participant with gout was individually matched based on age (±2 years), gender and cohort (CASHA, CASK, CASF) to four participants who had not consulted with gout in the same period.

### Radiographic assessment and scoring

Posterior-anterior (PA) plain radiographs of each hand were obtained from all clinic attenders in all three cohorts. Weight-bearing PA semi-flexed metatarsophalangeal, skyline and lateral knee views were obtained from CASK and CASHA participants and weight-bearing dorso-plantar and lateral views of each foot were obtained from CASF participants. Identical standardised radiographic protocols were used across the cohorts [6–8].

The Kellgren and Lawrence (K&L) system (0–4) was used to grade OA in 16 joints in each hand (interphalangeal joints (IPJs), metacarpophalangeal (MCPs), first carpometacarpal (CMC), trapezioscaphoid (TS)) [9]. K&L and Burnett atlases [10] were used to grade tibiofemoral (TF) and patellofemoral (PF) OA. The Menz atlas was used to grade osteophytes and joint space narrowing (JSN) (0–3) at five joints in each foot: the 1st MTPJ, the 1st and 2nd cuneometatarsal joints (CMJ), the navicular first cuneiform joint (N1stCJ) and the talonavicular joint (TNJ) [11]. Intra-rater and inter-rater reproducibility of OA at each joint site has been reported previously [12–14] and were found to be satisfactory.

### OA definitions

Hand OA and moderate-severe hand OA was defined respectively as the presence of radiographic OA K&L grade ≥ 2 and grade ≥ 3 affecting IPJs on at least two rays on each hand [5].

Radiographic PF joint OA was defined as K&L grade ≥ 2 on the skyline view and/or the presence of a definite superior or inferior patellar osteophyte (Burnett grade ≥ 1) on the lateral view [10] in either knee. Radiographic TF joint OA was defined as K&L grade ≥ 2 on the PA view and/or the presence of definite osteophyte (Burnett grade ≥ 1) on the posterior tibial surface on the lateral view [10] in either knee. Moderate to severe radiographic (K&L grade ≥ 3 and Burnett grade ≥ 3) TF and PF joint OA were also assessed.

Radiographic OA at each of the five foot joints was defined as a Menz grade ≥ 2 for osteophytes or JSN on either the dorso-plantar or lateral view in either foot, with severe OA categorised using a cut-off of Menz grade ≥ 3 [11]. Radiographic foot OA and moderate-severe OA were defined respectively as Menz grade ≥ 2 and grade ≥ 3 for osteophytes or JSN affecting one or more of the five joints in either foot [11].

### Chondrocalcinosis

Radiographic chondrocalcinosis (CC) is a common feature of calcium pyrophosphate dihydrate (CPPD) crystal deposition; therefore, radiology reports were screened for report of CC in order to assess possible misclassification bias.

### Statistical analysis

Crude odds ratio (OR) and 95 % confidence intervals (CI) were calculated between gout and the presence and severity of radiographic OA in the hand, knee and foot using conditional logistic regression models. ORs (95 % CI) were then adjusted for BMI and diuretic use in all three analyses and further adjustment made for hand pain in the hand analysis and knee pain in the knee analysis. Foot pain was not adjusted for in the foot analysis as all participants originated from CASF and had foot pain. Analyses were performed using SPSS Statistics version 20 and STATA version 12. All analyses were two tailed and a p value of ≤ 0.05 was considered statistically significant. Logistic regression models were tested and the assumptions for linearity and multi-collinearity were met.

## Results

**Fig. 1:**
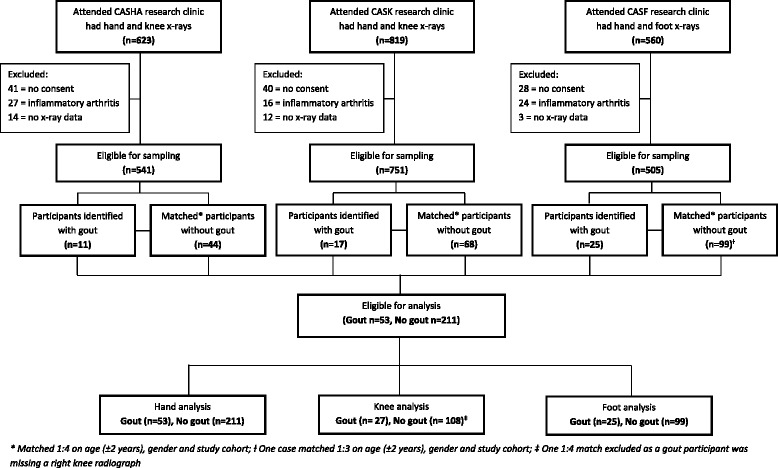
A flowchart showing participants eligible for sampling and analysis including exclusions

Following exclusions for non-consent for medical record review (*n* = 109), inflammatory arthritis (*n* = 67) and no radiographs (*n* = 29), 1797 participants were eligible for sampling (Fig. 1). Fifty-three participants with gout were identified (CASHA *n* = 11, CASK *n* = 17, CASF *n* = 25). Each was matched for age (±2 years) and gender to four individuals without gout from the same study cohort, with the exception of one gout individual from CASF for whom only three matched controls could be identified. Baseline demographic and clinical characteristics of the two groups are shown in Table 1. Mean BMI, diuretic use, ischaemic heart disease and hyperlipidaemia were higher in those with gout compared to those without gout. Knee and foot pain were more frequent and CC less frequent in those with gout than in those without.

**Table 1:** Baseline characteristics of participants with gout and participants without gout

	Gout		No gout	
	(*n* = 53)		(*n* = 211)	
Age: years, mean (SD)	66.4	(9.5)	66.6	(9.4)
Gender: Male	38	72 %	151	72 %
BMI: kg/m^2^, mean (SD)	31.3	(5.4)	29.2	(5.1)
BMI categories: <18.5 kg/m^2^	0	0 %	1	<1 %
18.5–24.9 kg/m^2^	1	2 %	36	17 %
25.0–29.9 kg/m^2^	29	55 %	93	44 %
≥ 30 kg/m^2^	23	43 %	80	38 %
Occupational class: managerial & professional	14	28 %	55	28 %
intermediate	12	24 %	37	19 %
routine & manual	24	48 %	103	53 %
Diuretic use	24	45 %	67	32 %
Co-morbidities: Hypertension	23	43 %	82	39 %
Ischaemic heart disease	11	21 %	29	14 %
Hyperlipidaemia	10	19 %	26	12 %
Diabetes mellitus	5	9 %	28	13 %
Chronic renal failure	2	4 %	1	<1 %
Knee pain	47	90 %	167	79 %
Hand pain	37	70 %	142	68 %
Foot pain	49	93 %	156	74 %
Chondrocalcinosis	2	4 %	26	12 %

*BMI* body mass index, *SD* standard deviation

### Hand analysis

Fifty-three participants with gout were compared with 108 matched participants without gout. No statistically significant association was observed between gout and radiographic hand OA on univariable analyses or after adjustment for BMI, diuretic use and hand pain. Although non-significant, participants with gout were more likely to have nodal hand OA and ≥ eight hand joints affected with moderate to severe radiographic hand OA compared to those without gout (Table 2).

**Table 2:** Crude and adjusted OR (95 % CI) for radiographic OA between participants with and without gout

Radiographic OA definition	Gout	No gout	Crude OR (95 % CI)	Adjusted OR (95 % CI)
Hand	*n* = 53	*n* = 211		^a^
Radiographic OA (K&L ≥ 2) affecting IPJs on at least two rays on each hand (%)	10 (19)	28 (13)	1.57 (0.69, 3.60)	1.46 (0.61, 3.50)
Moderate to severe radiographic OA (K&L ≥ 3) affecting IPJs on at least two rays on each hand (%)	3 (6)	12 (6)	1.00 (0.24, 4.11)	1.02 (0.22, 4.70)
Total number of joints affected by radiographic OA in the hand (K&L ≥ 2) [0–32]:				
0 (%)	17 (32)	62 (29)	1.00	1.00
1–2 (%)	15 (28)	62 (29)	0.91 (0.41, 2.03)	0.85 (0.37, 1.97)
3–7 (%)	13 (25)	65 (31)	0.72 (0.30, 1.75)	0.65 (0.26, 1.67)
≥ 8 (%)	8 (15)	22 (10)	1.39 (0.46, 4.15)	1.24 (0.38, 4.06)
Total number of joints affected by moderate to severe radiographic OA in the hand (K&L ≥ 3) [0–32]:				
0 (%)	34 (64)	134 (64)	1.00	1.00
1–2 (%)	8 (15)	46 (22)	0.76 (0.33, 1.77)	0.69 (0.29, 1.63)
3–7 (%)	7 (13)	25 (12)	1.17 (0.46, 2.98)	1.16 (0.45, 3.00)
≥ 8 (%)	4 (8)	6 (3)	3.48 (0.69, 17.72)	3.57 (0.62, 20.45)
Knee	*n* = 27	*n* = 108		^b^
Radiographic OA (K&L ≥ 2 or Burnett grade ≥ 1) affecting the TF joint in either knee (%)	11 (41)	49 (45)	0.82 (0.33, 1.99)	0.44 (0.15, 1.29)
Radiographic OA (K&L ≥ 2 or Burnett grade ≥ 1) affecting the PF joint in either knee (%)	20 (74)	76 (70)	1.22 (0.45, 3.29)	0.70 (0.22, 2.22)
Radiographic OA (K&L ≥ 2 or Burnett grade ≥ 1) affecting the TF and PF joints in either knee (%)	11 (41)	41 (38)	1.13 (0.46, 2.78)	0.57 (0.20, 1.65)
Radiographic OA (K&L ≥ 3 or Burnett grade 3) affecting the TF joint in either knee (%)	4 (15)	27 (25)	0.51 (0.16, 1.63)	0.28 (0.07, 1.09)
Radiographic OA (K&L ≥ 3 or Burnett grade 3) affecting the PF joint in either knee (%)	7 (26)	26 (24)	1.13 (0.39, 3.25)	0.58 (0.18, 1.86)
Radiographic OA (K&L ≥ 3 or Burnett grade 3) affecting the TF and PF joints in either knee (%)	3 (11)	10 (9)	1.22 (0.32, 4.71)	0.54 (0.12, 2.40)
Foot	*n* = 25	*n* = 99		^c^
Radiographic OA (Menz ≥ 2) affecting at least one of 1^st^ MTPJ, 1^st^ CMJ, 2^nd^ CMJ, N1^st^CJ and TNJ in either foot (%)	20 (80)	67 (68)	2.22 (0.69, 7.15)	2.16 (0.66, 7.06)
Severe radiographic OA (Menz ≥ 3) affecting at least one of 1^st^ MTPJ, 1^st^ CMJ, 2^nd^ CMJ, N1^st^CJ and TNJ in either foot (%)	7 (28)	21 (21)	1.46 (0.54, 3.94)	1.45 (0.53, 3.97)
Total number of joints affected by radiographic OA (Menz ≥ 2) [0–10]:				
0 (%)	5 (20)	32 (32)	1.00	1.00
1–2 (%)	10 (40)	46 (46)	1.64 (0.47, 5.72)	1.55 (0.44, 5.47)
≥ 3 (%)	10 (40)	21 (21)	3.86 (0.97, 15.31)	4.00 (0.99, 16.10)
Total number of joints affected by severe radiographic OA (Menz ≥ 3) [0–10]:				
0 (%)	18 (72)	78 (79)	1.00	1.00
1–2 (%)	7 (28)	21 (21)	1.46 (0.54, 3.94)	1.45 (0.53, 3.97)
Radiographic OA (Menz ≥ 2) affecting either 1st MTPJ (%)	13 (52)	37 (37)	1.90 (0.75, 4.78)	1.85 (0.73, 4.69)
Radiographic OA (Menz ≥ 2) affecting either 1st CMJ (%)	2 (8)	13 (13)	0.59 (0.13, 2.75)	0.54 (0.11, 2.56)
Radiographic OA (Menz ≥ 2) affecting either 2nd CMJ (%)	7 (28)	24 (24)	1.26 (0.45, 3.54)	1.34 (0.46, 3.91)
Radiographic OA (Menz ≥ 2) affecting either N1^st^CJ (%)	6 (24)	12 (12)	2.46 (0.78, 7.73)	2.60 (0.80, 8.46)
Radiographic OA (Menz ≥ 2) affecting either TNJ (%)	10 (40)	23 (23)	2.18 (0.87, 5.46)	2.24 (0.88, 5.70)

^a^Adjusted for BMI, diuretic use and hand pain. ^**b**^Adjusted for BMI, diuretic use and knee pain. ^**c**^ Adjusted for BMI and diuretic use. *K&L* Kellgren and Lawrence, *IPJs* interphalangeal joints, *OR* odds ratio, *CI* confidence interval, *TF* tibiofemoral, *PF* patellofemoral, *1st MTPJ* first metatarsophalangeal joint, *CMJ* cuneometatarsal joint, *N1stCJ* navicular first cuneiform joint, *TNJ* talonavicular

### Knee analysis

Twenty-seven participants with gout were compared to 108 matched participants without gout. One participant with gout had missing x-ray data for the right knee and so this individual and their four matched controls were excluded from this analysis. No statistically significant associations were observed between gout and radiographic knee OA on univariable analyses, or after adjustment but the magnitude of adjusted ORs suggested that participants with gout were less likely to have TF and PF OA than those without gout (Table 2).

### Foot analysis

Twenty-five participants with gout from CASF were compared to 99 matched participants without gout. No statistically significant associations were observed between gout and radiographic foot OA on univariable analyses or after adjustment but individuals with gout had 4 times the odds of having ≥ three foot joints affected with radiographic OA (Table 2). Although statistically non-significant, participants with gout were also more likely to have radiographic foot OA affecting at least one joint or a specific joint (1^st^ MTP, N1^st^CJ and TNJ) compared to those without gout (Table 2).

## Discussion

The rationale for investigating the association between gout and nodal OA was as an exemplar of generalised OA. However, no significant association was found between gout and radiographic hand OA. These findings are consistent with the only previous study examining the association between gout and hand OA, where nodal OA was found to be no more common in those with gout than those without gout [5]. There were several differences between that study and our current study. Although both studies used participants recruited from primary care, the previous study included younger adults (aged ≥30 years), based the diagnosis of gout on specialist clinical opinion, and used self-report instruments to assess clinical OA [5]. In contrast, in our study, OA was radiographically assessed; nevertheless, the definition of radiographic hand OA affecting at least two rays on each hand was comparable.

Although not statistically significant, there were findings in this study suggesting that participants with gout were more likely to have widespread or severe OA involvement of small joints in the hand and foot compared to those without gout but less likely to have TF or PF OA.

At the foot, individuals with gout were more likely to have foot OA, severe foot OA, and specific involvement of the 1st MTP, N1stCJ, TNJ and multiple joints compared to those without gout. 1st MTPJ involvement is not surprising as it is a common site for both OA and gout, and an association with OA has been suggested as a possible explanation for the striking predilection of gout for this joint [1, 15]. Previous research has also shown that the mid-foot region can be commonly affected by gout when clinical OA is evident [2], which is consistent with our observation that people with gout had a higher likelihood of mid-foot OA. The frequency of OA at individual foot joints may reflect the association of gout with generalised OA or that the pain associated with gout could alter biomechanics of the foot potentially predisposing to subsequent OA [2, 4].

This study, to our knowledge, is the first to examine the relationship between gout and radiographic OA across multiple joint sites. The study was undertaken in a primary care setting so the results are generalizable to most people with gout who are managed entirely in primary care. The major limitation of the study was inadequate statistical power, making it liable to a type II error. The study involved secondary analysis of data from three existing cohorts and therefore the number of available gout cases was limited. The sample population may also have differed from the general population as the frequency of radiographic OA was found to be considerably higher in these participants, who had all reported joint pain at the hand, knee or foot in the last year, than in other adult general populations. Therefore, an association between gout and OA may have been masked. A further caveat is the reliance on primary care records to diagnose gout, and whilst morbidity coding in the participating practices undergoes regular quality review [16, 17] and previous work has suggested that diagnosis of gout by GPs is reasonably accurate [18], there is still a risk of misclassification bias. Synovial fluid MSU crystal identification is the gold standard for the diagnosis of gout [19], however, this is rarely performed in primary care. It is, however, reassuring that only a small minority of participants had radiographic CC.

## Conclusion

In summary, no statistically significant association was observed between gout and hand, knee and foot OA, however there were findings to suggest that people with gout were more likely to have OA affecting the small joints of the hands and feet but less likely to have large joint OA at the knee. Further research is needed to understand the relationship between gout and OA, using robust case definitions based on crystal identification and imaging. Such studies will provide important insights into the aetiology, co-occurrence and clinical presentation of both conditions.

## Ethics and consent to participate

Ethical approval was obtained for all three cohorts (North Staffordshire LREC (1430); Coventry REC (10/H1210/5). All participants provided written informed consent and were also asked for consent to review their medical records.

## Consent to publish

Not applicable as no identifying personal information is being published in this manuscript.

## Availability of data and materials

Requests for further detail on the dataset and queries relating to data sharing arrangements may be submitted to Edward Roddy, e.roddy@keele.ac.uk. Participants were not asked for informed consent for data sharing although the presented data are anonymised and risk of identification is low.

## Abbreviations

BMI: body mass index
CASF: clinical assessment study of the foot
CASHA: clinical assessment study of the hand
CASK: clinical assessment study of the knee
CC: chondrocalcinosis
CI: confidence interval
CMC: carpometacarpal
CMJ: cuneo-metatarsal joint
CPPD: calcium pyrophosphate dihydrate
DIP: distal interphalangeal
GP: general practitioner
IPJ: interphalangeal joint
JSN: joint space narrowing
K&L: kellgren and lawrence
MCP: metacarpophalangeal
MSU: monosodium urate
MTPJ: metatarsophalangeal joint
N1^st^CJ: navicular-first cuneiform
OA: osteoarthritis
OR: odds ratio
PA: posteroanterior
PF: patellofemoral
PIP: peripheral interphalangeal
TF: tibiofemoral
TNJ: talo-navicular joint
TS: trapezioscaphoid
